# The prevalence of depression in rheumatoid arthritis in China: A systematic review

**DOI:** 10.18632/oncotarget.17323

**Published:** 2017-04-21

**Authors:** Xin Fu, Zhi-Jun Li, Chun-Jun Yang, Liangshu Feng, Lemeng Sun, Yang Yao, Yu-Ting Huang

**Affiliations:** ^1^ Department of Orthopaedics, Tianjin Hospital, Tianjin, China; ^2^ Department of Orthopaedics, Tianjin Medical University General Hospital, Tianjin, China; ^3^ Department of Nursing, Tianjin Medical University General Hospital, Tianjin, China; ^4^ Department of Neurology and Neuroscience Center, First Hospital of Jilin University, Changchun, China; ^5^ Cancer Center, First Hospital of Jilin University, Changchun, China; ^6^ Department of Neurology, Tianjin Medical University General Hospital, Tianjin, China; ^7^ Children's Research Institute, Children's National Medical Center, Washington, DC, USA

**Keywords:** depression, rheumatoid arthritis, prevalence, meta-analysis

## Abstract

This systematic review is to explore the prevalence of depression in patients with rheumatoid arthritis (RA) in China. Articles of prevalence rates for depression in adult RA patients published before October 2015 were identified from PubMed, Embase, The Cochrane Library, CNKI, CBM, VIP, and Wanfang database and other internet databases. Relevant journals and the recommendations of expert panels were also searched manually. Two independent reviewers searched and assessed the literature. Therelevant data were applied with Meta-Analyst 3.13 software, and the forest plot and funnel plot were performed. 21 studies with a total of 4447 patients were selected to be enrolled in this study. The prevalence of depression by analyzing the effect size was 48% [95% CI (41%, 56%)]. The prevalence of minor depression and dysthymic disorder was 30% [95%CI (23%, 38%)], and the moderate or major depression was 18% [95%CI (11%, 29%)], respectively. Subgroup analysis showed that the depression rate of female RA patients was higher than male. The depression rate in the central and western areas were higher than that of the eastern region of China, the prevalence level estimated by the Geriatric Depression Scale (GDS) was higher than estimated by other tools. Sensitivity analysis showed that the pooled effect size had good stability and reliability, To be conclusive, the prevalence rate of depression in RA patients is 48%, which suggesting that medical staff should pay more attention to depression in adult patients with RA.

## Level of evidence III

Rheumatoid arthritis (RA) is a chronic disease with an unknown etiology, presenting as systemic inflammatory autoimmune diseases, characterized by joint pain, swelling, stiffness, and deformation. At the early stage, RA symptoms mainly include fatigue, lack of appetite, low-grade fever, and joint aches; at the late stage, the symptoms of joint, including stiffness, deformity, and even loss of function. These clinical manifestations may subsequently lead to psychological distress [1]. Depression is highly prevalent in individuals with RA, with some studies reporting that the prevalence rates of depression are up to 41.5% [2], higher than the general population [3], even higher than those patients with diabetes [4], Parkinson [5], and cancer [6]. Depression in RA is associated with increasing pain [7], fatigue [8], physical disability [9], co-morbidities, health service utilization [10], mortality [11], suicide risk [12], and healthcare costs [13]; decreased in quality of life [14], treatment compliance, and work productivity [15].

Nearly 1% of the populations throughout the developed countries are affected by RA [10]. Previous studies have attempted to use some data to examine the strength of the associations between depression and RA. Different studies on RA patients with depression in China reported the prevalence estimates for depression range greatly from 8% to 78% [16–33]. The wide range in the prevalence of depression in the clinical studies of RA is likely due to the difference of study quality, variations in definitions of depression, and the methods used for measuring the depressive symptoms. Matcham *et al*. had provided pooled prevalence rates of depression in patients with RA in the world range [34]. However, according to the difference of religious belief, geographic distribution and socio-economic status in China, the prevalence estimates for depression is different comparing with the results in other countries. Despite, the importance of depression with RA patients, the aim of developing this systematic review study by performing a meta-analysis of the available data from previous studies is to comprehensively investigate pooled prevalence estimates of RA patients with depression in China.

## RESULTS

### Literature search results

**Figure 1:**
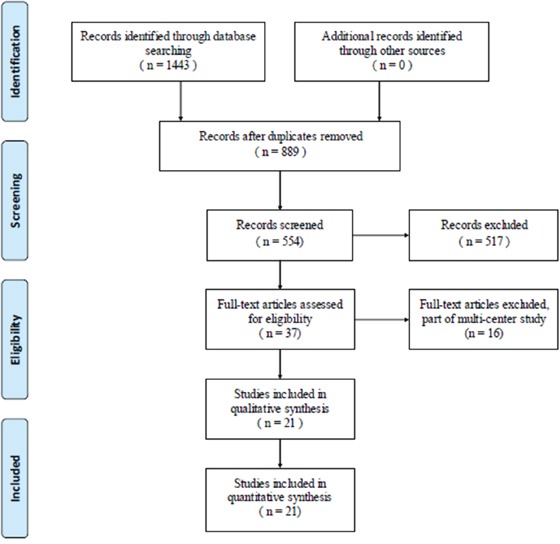
Flow diagram of the literature search and selection process

The search yielded 1443 journal articles, all duplicate articles were removed. 554 potentially eligible studies subjected to further screening. By reading the title and abstract, 517 studies did not meet our selection criteria. After review of the full text, 16 articles were ruled out. Finally, 21 studies were included with a total of 4447 individuals in the analysis (Figure 1).

### Study characteristics

Twenty-one studies with a total of 4447 individuals were included in the analysis. The characteristics of included studies are summarized in Table 1. 2580 individuals were reported as RA patients with depression. The median number of participants per-study was 212, ranging from 30 to 2156. To further characterize the range of depression or depressive symptom prevalence estimates identified by these methodologically diverse studies, three studies [18–19, 28] did not mention the method sused to identify patients with depression, two studies [22, 31] used the Hamilton Depression Scale (HAMD) to identified, and the remaining 16 studies were using the Self-rating Depression Scale (SDS) to evaluate. Nine studies [18–19, 22, 26, 29, 31, 35–36] have demonstrated the rate of major depression and minor depression and dysthymic disorder, including 337 individuals with minor depression and dysthymic disorder, and 279 individuals with major depression.

**Table 1:** Summary of clinical studies characteristics and prevalence rates for depression in RA patients

Study	Duration	Province	Sample size	NO. of A	Prevalence	NO. of B	NO. of C	Tools
Wu XM	2002-2004	Jiangsu	86	63	73%	NS	NS	SDS
Li H	2010	Anhui	173	127	73.4%	76	51	SDS
Yao XM	2008-2010	Guizhou	258	181	70.16%	89	92	SDS
Li CF	2008	Henan	40	12	30%	NS	NS	SDS
Ru HY	2010-2011	Guangdong	38	10	26.3%	NS	NS	SDS
Yin PH	2000-2001	Beijing	30	22	73.3%	11	11	NS
Wu M	2009-2010	Guangdong	160	125	78.1%	67	NS	NS
Dong LX	2011-2012	Hunan	169	64	37.8%	NS	NS	SDS
Wang YG	2010	Guangdong	150	12	8%	8	3	SDS
Yin GF	2009-2010	Shangdong	160	56	35%	33	19	HAMD
Huang JL	2004-2005	Guangdong	60	18	30%	NS	NS	SDS
Liu J	2005-2006	Anhui	136	84	61.76%	NS	NS	SDS
Zheng ZJ	2006-2007	Anhui	100	53	53%	39	11	SDS
Guo JB	2011-2012	Hebei	2156	1433	66.5%	NS	NS	SDS
Zhang H	2008-2009	Hubei	86	35	34.9%	NS	NS	NS
Li QP	2004	NS	58	27	27.6%	16	NS	SDS
Wang Y	2009-2011	Shangxi	201	87	43.3%	NS	NS	SDS
Chen X	2007-2011	Beijing	112	62	55.4%	38	15	HAMD
Chen Q	2007-2010	Sichuan	125	49	39.2%	NS	NS	SDS
Jiang LD	1997-1998	Shanghai	95	41	43.2%	NS	NS	SDS
Wang JL	2005-2006	Hebei	54	19	35.2%	NS	NS	SDS

NS: no stated; A: patients with depression; B: patients with mild depression; C: patients with moderate or severe depression; SAS: Self-Rating Depression Scale; HAMD: Hamilton depression Rating Scale.

### Quality assessment

According to the quality assessment tool (JBI standard), the overall quality of all 21 articles was generally good. Eighteen studies [16–20, 23–32, 35–37] scored 14/20 or higher, and four articles [19, 21–22, 33] scored 13/20 due to not describing in detail the method of random sampling, the inclusion and exclusion criteria, sample characteristics, and the lack of checking information.

### Results of the meta-analysis

#### Prevalence of depression

**Figure 2:**
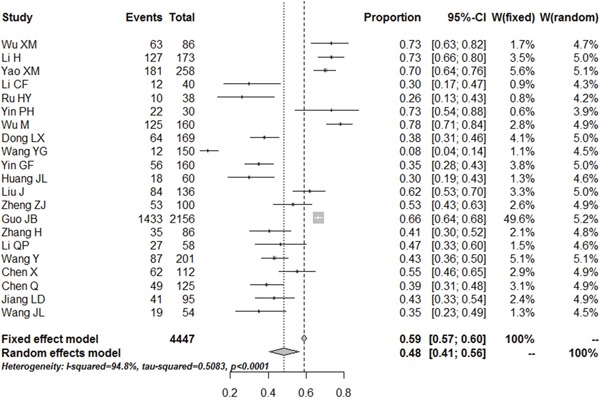
Meta-analysis of the prevalence rates for depression in RA patients in China

The meta-analytical pooled prevalence of depression (Figure 2) according to the diagnostic criteria of HAMD or SDS was 48% [95% CI (41%, 56%)] with moderate heterogeneity (I^2^=94.8%). The results of publication bias showed in (Supplementary Figure 1). Visual inspection of the funnel plot of studies reporting on depression or depressive symptoms revealed significant asymmetry, which indicating possible publication bias.

#### Prevalence of minor depression and dysthymic disorder

**Figure 3:**
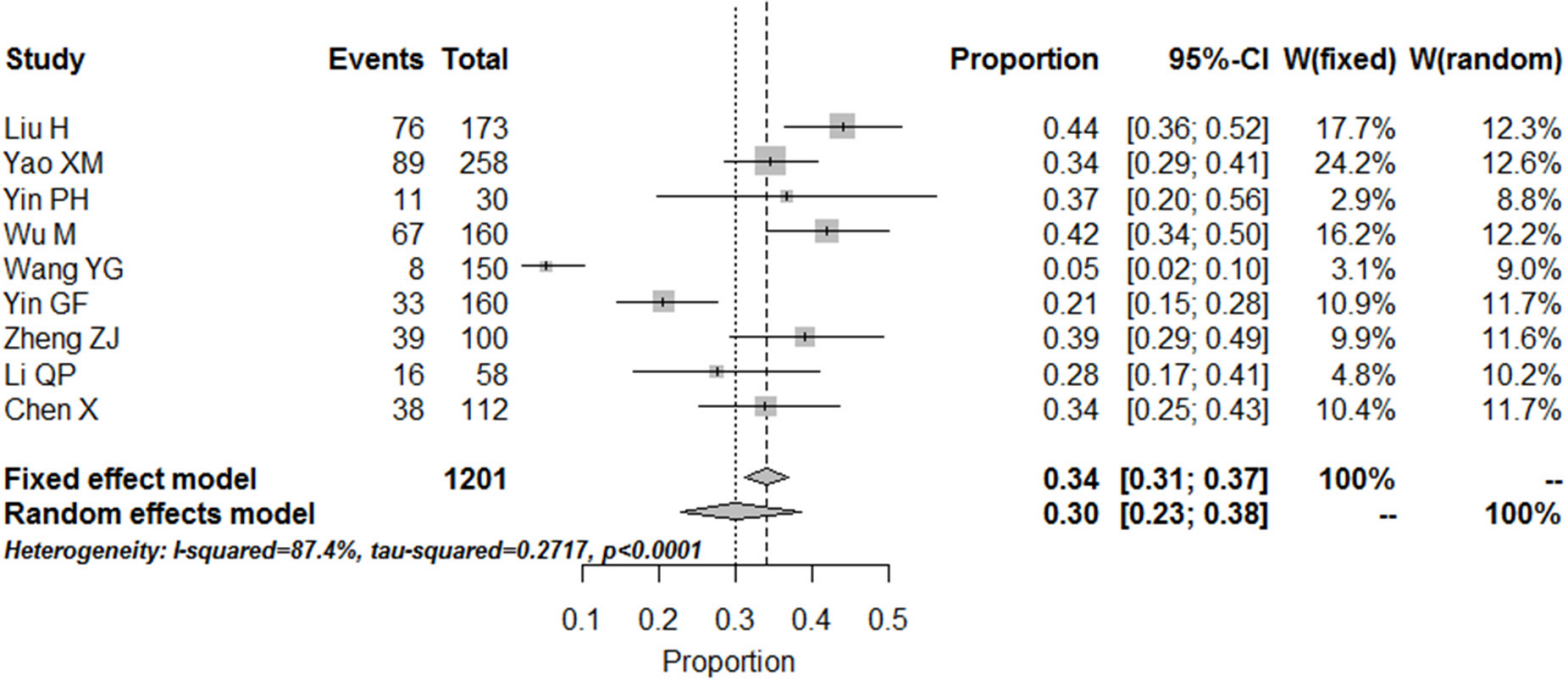
Meta-analysis of the prevalence rates for minor depression in RA patients in China

Nine articles evaluate the prevalence of minor depression and dysthymic disorder by diagnostic criteria of SDS, the analysis indicating a pooled prevalence level of 30% [95% CI (23%, 38%)], with moderate heterogeneity (I^2^ = 87.4%) (Figure 3). Visual inspection of the funnel plot of studies suggested possible publication bias (Supplementary Figure 2).

#### Prevalence of major depression

**Figure 4:**
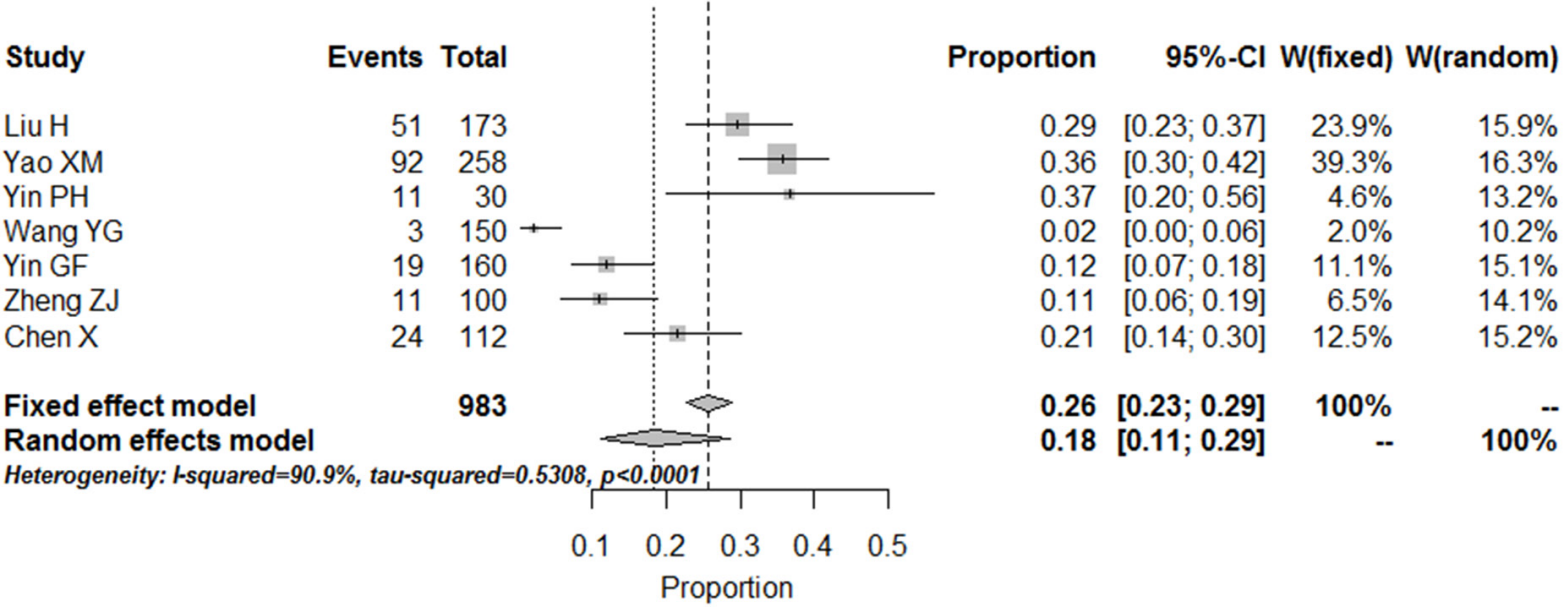
Meta-analysis of the prevalence rates for moderate and major depression in RA patients in China

Nine articles also evaluate the prevalence of major depression according to the SDS, indicating a pooled prevalence level of 18% [95% CI (11%, 29%)] (Figure 4). Visual inspection of the funnel plot of studies reporting on depression or depressive symptoms revealed significant asymmetry, which indicating possible publication bias (Supplementary Figure 3).

#### Sensitivity analysis and subgroup analysis

After removing two studies using HAMD (as diagnostic criteria) to identified the level of depression, pooled effects size is 49% [95%CI (40%, 57%)]. The results of sensitivity analysis showed that the main effect size was of stability and reliability. The subgroup analyses were conducted according to sample size, overall quality and publication year. The subgroup analysis showed that the depression rate of female RA patients was higher than male. The depression rate in the central and western areas was higher than that of the eastern region of China, the prevalence level estimated by the GDS was higher than estimated by other tools, However, more recent publications tended to yield higher prevalence estimates.

## DISCUSSION

Depression is highly prevalent in RA patients. Estimates varied according to the way in which depression was measured, but this study used the Meta-Analyst 3.13 software to measure the prevalence of depression in RA patients in China, which the result suggests that the depressive symptoms present in 48% [95% CI (41%, 56%)] in China, closing to the median 41.2% in all 21 studies. The minor depression and dysthymic disorder is present in 30% [95% CI (23%, 38%)] and moderate to major depression is present in 18% [95% CI (11%, 29%)]. One previous meta-analysis published in 2013 showed that significant depressive symptoms presented in 38.8% using the PHQ-9 and between 14.8% and 48% using the HADS [38]. Compared with the results of this study, the prevalence estimates for depression is higher in our study. The cause of this difference is likely to depend on religious belief, geographic distribution and socio-economic status, which need to be explored further.

The results of the prevalence rates of depression in RA differ significantly between China and other countries, mainly for the following reasons: firstly, most self-rating scales are chosen as the assessment tools for depression because they are cheaper and can be completed quickly, which could probably be influenced by subjective factors of patients. Secondly, it is likely to produce inaccurate results due to the lack of sample size or high-quality studies;finally, a diversity of studies varied widely in terms of the baseline, such as the age proportion, complication, can be considered as interference factors [39].

Various assessment questionnaires were used to measure the level of depression, such as the Patient Health Questionnaire (PHQ) [40], the Hospital Anxiety and Depression Scale (HADS) [41], Beck depression inventory (BDI) [42], Self-Rating Depression Scale (SDS) [43] and GDS [44]. However, the screening questionnaire of Hamilton Depression Rating Scale (HAMD) and SDS are the most commonly used in China. In this systematic review, all studies used SDS or HAMD to ascertain the level of depression. As a self-report screening tool, SDS is commonly used to assess the level of depression of patients, and its reliability and validity have been examined in China [45]. It includes 20 items, with each subscale rated on a four-point scale and scored from 1 to 4 (never or occasionally, sometimes, often and most of the time). Total scores are calculated by summing the items on each subscale, and the standardized score is the total score times 1.25, a total standard score of 53 was set as a cut-off point of depression. Scores between 53 and 62 represent minor depression and dysthymic disorder; 63 to 72 suggest ‘moderate depression’ and above 72 indicate ‘major depression’ [46–47]. HAMD is a validated method for measuring the level of depression. It consists of 24 items, in which 14 items is scored from 0 to 4 and 10 items is scored from 0 to 2, resulting in a total score of 0 to76. According to the classification criterion: the level of depression is divided into no depression (0-7); minor or moderate depression (20-34); major depression (above 35) [48]. Therefore, different screening tools have different cut-point to assess the level of depression, and prevalence estimates are often relying on predefined thresholds, therefore, the prevalence rates of depression according to these different tools may be inaccurate. In this review, the pooled effects size is 48% [95% CI (41%, 56%)] after removing two studies using HAMD, indicating that the main effect size was of stability. The high rates of depressive symptomatology detected through the screening tools could be due to the overlap between the somatic symptoms of depression and symptoms of RA. Symptoms frequently associated with depression (such as fatigue and reduced sleep quality) may be experienced by RA patients regardless of whether depressive symptoms are present or not.

According to sensitivity analysis and publication bias, the results of this study showed that the prevalence estimates of depression were stable, significant publication bias is presented in the prevalence estimates for minor and major depression because of insufficient sample size. More high-quality researches are needed in the future.

There are some limitations in this study, which needed to be addressed: firstly, the data were derived from studies that had different designs, screening instruments, and trainee demographics. The substantial heterogeneity among the studies remained largely unexplained by the variables inspected. Secondly, because the studies were heterogeneous with respect to screening inventories and populations, the prevalence of major depression could not be determined. Finally, many subgroup analyses relied on unpaired cross-sectional data collected at different medical centers, which may cause confounding.

## CONCLUSION

The result of this meta-analysis is almost consistent with national epidemiological survey of RA patients in China, indicating the result is reliable, which provides key parameter for making strategies to care for the patients with rheumatoid arthritis. Furthermore, efforts are continually needed to reduce barriers to mental health services, including addressing the stigma of depression.

## MATERIALS AND METHODS

### Search strategy

For our systematic review, we looked at all English and non-English academic articles identified from different electronic databases, including MEDLINE, EMBASE, Chinese journal full-text database (CNKI), Chinese biomedical database (CBM), Chinese science and technology periodical database, and Wanfang database. The search strategy is presented in Figure 1. In addition, the Google scholar search engine was searched manually using the same search terms to seek further relevant studies that may have been missed in the database search. We used the keywords ‘Rheumatoid arthritis’ and ‘depression’ and ‘China’. Broad MeSH terms and Boolean operators were selected for each database search. The literature search was updated on October 2015. The reference lists of all the full-text papers were examined to identify any initially omitted studies. We made no restrictions on the language of the publications [38].

### Data extraction and quality assessment

The selection criteria used to evaluate the association between rheumatoid arthritis and depression, each article using a standardized form: the age of RA patientsis above 18, reported a prevalence level or incidence of depression in RA patients, the sample size was more than 30, the cross-sectional observational study, baseline data including the level of depression.

For each eligible study, two of the reviewers (Li Z.J. and Fu X.) extracted all the relevant data independently. Any disagreement was resolved by discussion when no consensus could be achieved, the third reviewer (Huang Y.T.) was the adjudicator and made the final decision. Whenever necessary, we contacted the authors of the studies for the missing data or further information. The following data were extracted: the secondary sources including the review, the error in research design or statistical method, studies in special population, such as pregnant women with RA, children with RA. We assessed trial quality using the JBI (Joanna Briggs Institution) critical appraisal score that is reliable. The quality assessment tool consists of a total of 10 items with each item scored from 0 to 20. Total scores are calculated by summing the items on each subscale, and the article was regarded as good quality if the score was above 14 [49].

### Outcome measures

Outcomes were divided into mild, moderate or major depression according to the screening tool of Hamilton depression scale (HADS) or self-rating depression scale (SDS).

### Statistical analysis

The Meta-Analyst 3.13 software was used to undertaken meta-analysis. The ratio and 95% confidence interval (CI) from each study were calculated. The heterogeneity was assessed using I^2^, an I^2^ value of 50% was considered to indicate substantial heterogeneity. The origins of heterogeneity, if present, were analyzed according to differences in methodological quality and the characteristics of the participants. Heterogeneity was found to be moderately high between studies, and therefore random effects meta-analyses with 95% CIs were conducted [50–51]. When the data allowed, the authors of this paper performed subgroup analysis and sensitivity analyses. Funnel plots were produced to explore the possibility of publication bias.

## SUPPLEMENTARY FIGURES


