# Supplementary material for: The prevalence of depression in rheumatoid arthritis in China: A systematic review
Source: Oncotarget. 2017 Apr 21;8(32):53623–30. doi: 10.18632/oncotarget.17323 (PMC5581135; doi:10.18632/oncotarget.17323)
Supplement: Supplementary file 1 [file oncotarget-08-53623-s001.pdf]

The prevalence of depression in rheumatoid arthritis in China: A systematic review

SUPPLEMENTARY FIGURES

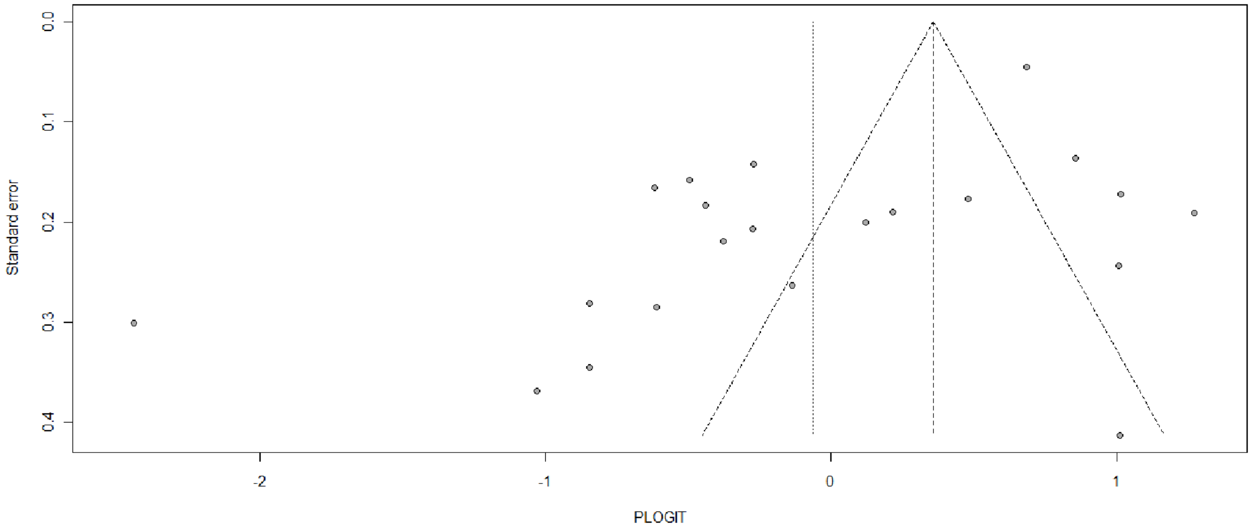

Supplementary Figure 1: Meta-analysis of publication bias of RA patients with depression in China.

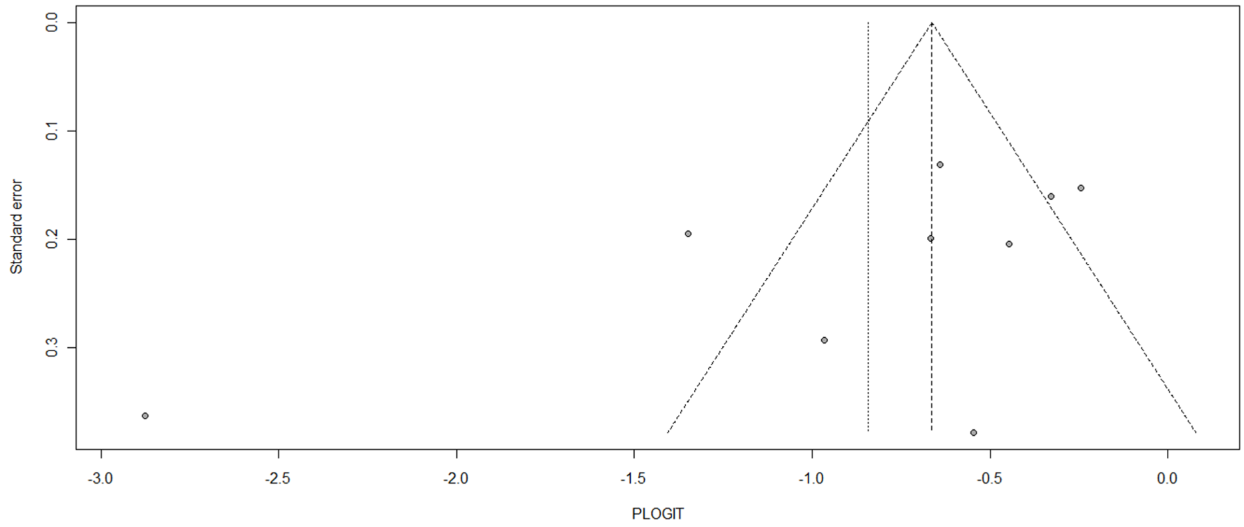

Supplementary Figure 2: Meta-analysis of publication bias of RA patients with mild depression in China.

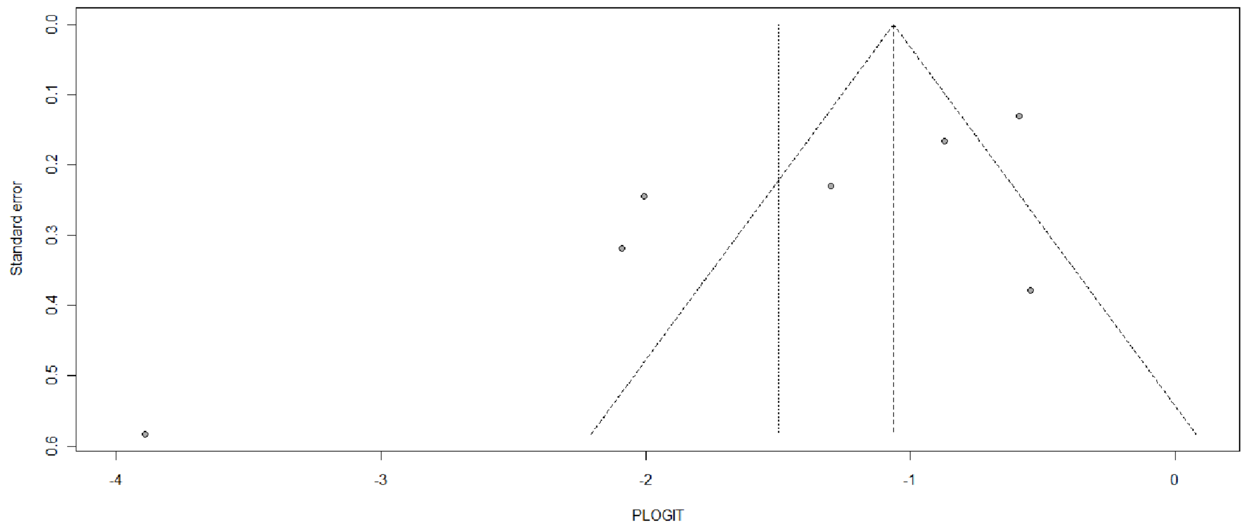

Supplementary Figure 3: Meta-analysis of publication bias of RA patients with moderate and severe depression in China.
